# Five-Year Mortality of Surgical and Transcatheter Aortic Valve Replacement in the Real-World Scenario: A Systematic Review and Meta-Analysis of Propensity Score Matching Studies

**DOI:** 10.21470/1678-9741-2024-0048

**Published:** 2025-04-15

**Authors:** Mateo Marin-Cuartas, Bianca Dalbesio, Francesco Pollari, Matteo Scarpanti, Amedeo Anselmi, Manuela de la Cuesta, Miguel Sousa Uva, Jean-Philippe Verhoye, Francesco Musumeci, Fabio Barili, Alessandro Parolari

**Affiliations:** 1 University Department of Cardiac Surgery, Leipzig Heart Center, Leipzig, Germany; 2 Department of Cardiac Surgery, S. Croce Hospital, Cuneo, Italy; 3 Cardiac Surgery, Klinikum Nürnberg-Paracelsus Medical University, Nuremberg, Germany; 4 University Cardiac Surgery Unit, IRCCS Policlinico San Donato, San Donato, Italy; 5 Department of Thoracic and Cardiovascular Surgery, University Hospital of Rennes, Rennes, France; 6 Department of Cardiac Surgery, Hospital Santa Cruz, Carnaxide, Portugal; 7 Department of Cardiac Surgery and Physiology, Porto University Medical School, Porto, Portugal; 8 Department of Cardiac Surgery, ISMETT, Palermo, Italy; 9 University Cardiac Surgery Unit, IRCCS Ospedale Galeazzi Sant’Ambrogio, Milan, Italy; 10 Department of Epidemiology, Harvard T.H. Chan School of Public Health, Boston, Massachusetts, United States of America; 11 Department of Biomedical and Clinical Sciences, University of Milan, Milan, Italy

**Keywords:** Transcatheter Aortic Valve Replacement, Aortic Valve, Meta-Analysis, Systematic Review

## Abstract

**Introduction:**

Randomized controlled trials (RCTs) provide evidence of efficacy, while real-world data (RWD) demonstrate effectiveness in real-world practice. We designed a systematic review and meta-analysis of reconstructed time-to-event (RTE) data from propensity score matching studies comparing transcatheter aortic valve implantation (TAVI) and surgical aortic valve replacement (SAVR) to compare their effectiveness and evaluate the generalizability of TAVI indications.

**Methods:**

Systematic review of literature between 2007 and 2023 including propensity score matching studies comparing TAVI or SAVR that reported at least one-year Kaplan-Meier curves of endpoints.

**Results:**

Twenty-one studies were included (39538 participants). TAVI shows a higher all-cause mortality (hazard ratio [HR] 1.41; 95% confidence interval [CI] 1.34-1.47, P-value < 0.001), with a significant heterogeneity. The analysis of HR trend over time shows that TAVI superiority is limited to the first month with a steep reversal afterwards, when SAVR becomes clearly superior. All-cause mortality is significantly higher in TAVI in low-risk (HR 1.35; 95% CI 1.08-1.69, P-value < 0.001) as well as in intermediate (HR 1.73; 95% CI 1.35-2.22, P-value < 0.001) and high-risk (HR 1.61; 95% CI 1.38-1.88, P-value < 0.001) patients. The HR trend in the subgroups of risk confirms the data from the whole mixed population.

**Conclusion:**

In a real-word setting, TAVI is associated with higher incidence of all-cause death and maintains a survival benefit only in the first month after implantation. These results show that TAVI effectiveness may not reflect the efficacy demonstrated by RCTs and pose a threat to their external validity.

## INTRODUCTION

**Table:** 

Abbreviations, Acronyms & Symbols				
BEV	= Balloon-expandable valve		NA	= Not applicable
BMI	= Body mass index		PCI	= Percutaneous coronary intervention
CABG	= Coronary artery bypass grafting		RCTs	= Randomized controlled trials
CI	= Confidence interval		RTE	= Reconstructed time-to-event
COPD	= Chronic obstructive pulmonary disease		RWD	= Real-world data
EuroSCORE	= European System for Cardiac Operative Risk Evaluation		RWE	= Real-world evidence
FDA	= Food and Drug Administration		SAVR	= Surgical aortic valve replacement
HR	= Hazard ratio		SEV	= Self-expanding valve
ICD	= Implantable cardioverter defibrillator		STS-PROM	= Society of Thoracic Surgeons Predicted Risk of Mortality
KM	= Kaplan-Meier		TAVI	= Transcatheter aortic valve implantation
MIC	= Minimally invasive cardiac surgery			

The development and diffusion of the transcatheter approach for the treatment of aortic valve disease have been driven by an unprecedented amount of randomized controlled trials (RCTs), most of them sponsored by companies that addressed recent guidelines and lead to a broadening of the indications for the transcatheter approach to include lower categories of risk^[1-9]^. Transcatheter aortic valve intervention (TAVI) is nowadays more common than surgical aortic valve replacement (SAVR), partially because current evidence has influenced patients’ wishes and the definition of risk profile has been overcome by the concepts of life expectancy, frailty, and specific transcatheter and surgical risk factors.

In evidence-based medicine, RCTs provide the highest hierarchical level of evidence based on a single experiment. However, randomization allows control for confounding on admission but does not protect from biases other than non-random allocation, which can pose a serious threat to internal validity^[10]^. Moreover, RCTs may lead to critical issues in the external validity, as they require strict inclusion and exclusion criteria, thus limiting the generalizability of the results to broader population^[11,12]^. The expansion of an intervention program that benefits a specific subgroup of patients to a broader population without evidence, named indication creep, is evident in RCTs comparing TAVI and SAVR, particularly in low-risk studies^[13]^. These RCTs are designed in very selected cohorts, as demonstrated by their several exclusion criteria, and do not represent the entire population from which they are extrapolated. The mean age of the low-risk trial is exemplificative, being over 74 years for all low-risk RCTs cohorts^[5-7]^; nonetheless, current American guidelines have generalized their recommendation to all low-risk patients over 65 years^[14]^.

The void of information in RCTs on the most vulnerable patients and the whole population may be implemented integrating real-world data (RWD) in the decision making^[12]^. RCTs provide evidence of efficacy, while RWD produces evidence of effectiveness in real-word practice settings and can identify previously unrecognized aspects related to treatment, although it is unreliable for assessing causal relationship and intrinsically have a higher risk of bias^[15]^. Regulators are paying growing attention to the complimentary information of real-world evidence (RWE), and the United States of America’s Food and Drug Administration (FDA) developed a framework to evaluate the potential use of RWE to help support the approval of a new indication for a previously approved drug or to help support post-approval drug study requirements^[16]^. RWE of TAVI *vs.* SAVR may furnish integrative data on effectiveness to support the generalization of TAVI indication beyond the strict exclusion and inclusion criteria of RCTs. Advances in methodologies for non-randomized studies, as well as statistical tools for minimizing the effects of confounders such as balancing methods, have contributed to ameliorating RWE’s quality and reliability, although not all sources of bias can be removed^[11]^.

Hence, we designed a systematic review and meta-analysis of reconstructed time-to-event (RTE) data from propensity score matching studies comparing TAVI and SAVR to compare their effectiveness on mid-term all-cause mortality to evaluate the generalizability of TAVI indication.

## METHODS

### Search Strategy and Selection Criteria

The study protocol adheres to the Preferred Reporting Items for Systematics Reviews and Meta-analyses (or PRISMA) statement^[17]^. The protocol has been registered in PROSPERO (CRD42023455630).

A systematic review of the literature was performed by two independent researchers to identify eligible studies published between January 1^st^, 2007 and May 31^st^, 2023, in MEDLINE, Embase, and the Cochrane Central Register of Controlled Trials (or CENTRAL). The inclusion criteria were: 1) propensity score matching studies comparing TAVI or SAVR; 2) at least one-year follow-up; 3) the full report of Kaplan-Meier (KM) curves of all-cause mortality in Text or Appendix, including the correct report of patients at risk and perioperative mortality. The meta-analysis’ endpoint was death from any cause at follow-up. The hazard ratio (HR) was considered the effect size. HRs were estimated from pooled RTE data with Cox models and fully parametric models. For studies on the same population, we selected the longest available follow-up report.

### Data Extraction and Analysis

Two independent investigators (BD and MS) identified trials that fulfilled the pre-specified inclusion criteria. Eligible trials were then reviewed in duplicate, and disagreement was solved by a third investigator (FB). Extracted data from the Text and Appendix were trial characteristics, patients' baseline data and comorbidities, device type, and implantation access.

In meta-analysis of aggregated time-to-event data across trials, the appropriate effect measurement is the HR^[18,19]^. The HRs were derived using time-to-event data reconstructed from digitally captured KM curves. Time-to-event data was extracted at the individual level from KM graphs, employing a dedicated software (Plot Digitized 2.6.2 for Macintosh) to digitize KM curves and a KM-data reconstruction algorithm coded in R for estimating the individual patient data as previously described^[18-20]^. HRs were estimated from pooled RTE data with both semi-parametric and fully parametric models.

### Risk of Bias and Quality Assessment

The risk of bias among included studies was estimated by two Authors (FB, AP) using the ROBINS-I tool for non-randomized studies^[21]^.

### Statistical Analysis

The cumulative incidence of outcomes at follow-up in the two treatment arms was evaluated with KM estimates. Unadjusted HRs in the pooled dataset were estimated with grouped frailty semi-parametric (Cox) model, accounting for heterogeneity among trials with a random-intercept parameter, as previously described^[22]^. Proportionality of hazards of the Cox models was checked with the Grambsch-Therneau test and diagnostic plots based on Shoenfeld residuals. We planned to perform landmark analysis for evidence of non-constant proportional hazards from test results or visual inspection of KM curves. The time-varying HR of endpoints for TAVI *vs.* SAVR was modeled with fully parametric generalized survival models (Royston-Parmar models) with baseline smoother and time-varying variables based on b-splines.

Quality assessment of RTE data was performed graphically checking the derived KM curves with the original ones. Moreover, the accuracy was evaluated by comparing the estimated and reported (when available) HRs. We assessed potential publication bias with visual interpretation of funnel plot.

Analyses were performed with R language (R 4.2.0; R Development Core Team [2022]. R: A language and environment for statistical computing. R Foundation for Statistical Computing, Vienna, Austria).

### Ethic Statement

This meta-analysis study is exempt from ethics approval as we collected and synthesized aggregate data published from previous clinical trials in which informed consent has already been obtained for the individual data by the trial investigators.

## RESULTS

### Baseline Characteristics and Risk of Bias

After literature search, eligibility evaluation, and duplicates’ exclusion, 52 studies were checked for further assessment. We excluded 31 studies that did not fulfill inclusion criteria. Twenty-one studies fulfilled the pre-specified inclusion criteria and were included in the meta-analysis^[23-43]^.

Table 1 reports baseline characteristics of the study groups. Overall, 39538 patients underwent TAVI (n=19661) or SAVR (n=19877). Most of the studies were performed on a cohort of mixed risk profile (34840 patients) - 1746 low-risk, 1082 intermediate risk, and 1870 high-risk patients. In the study cohort, both balloon-expanding and self-expanding TAVI devices were under study. The TAVI approaches were different; however, the most common access was transfemoral.

**Table 1:** Study groups’ baseline characteristics.

Study	Papadopoulos et al.^[35]^	Johansson et al. ^[32]^	Sponga et al.^[38]^	Brennan et al.^[27]^	Armoiry et al.^[24]^	Barbanti et al.^[25]^	Schaefer et al.^[37]^	Virtanen et al.^[42]^	Tzamalis et al.^[40]^	Takeji et al.^[39]^	Muneretto et al.^[34]^	Ferrara et al.^[31]^	Beyersdorf et al.^[26]^	Brìzido et al. ^[28]^	Chung et al.^[29]^	Santarpino et al.^[36]^	Deharo et al.^[30]^	Vilalta et al.^[41]^	Alperi et al.^[23]^	Kowalówka et al.^[43]^	Kolar et al.^[33]^	
Treatment group	Redo-TAVI Redo-SAVR	TAVI SAVR	TAVI SAVR	TAVI SAVR	TAVI SAVR	TAVI SAVR	TAVI SAVR	TAVI SAVR	TAVI SAVR	TAVI SAVR	TAVI SAVR	TAVI SAVR	TAVI SAVR	TAVI SAVR	TAVI SAVR	TAVI SAVR	TAVI SAVR	TAVI SAVR	TAVI + PCI SAVR + CABG	TAVI SAVR	TAVI SAVR	
Trial's characteristics																						
Year	2014	2016	2017	2017	2018	2019	2019	2020	2020	2020	2020	2021	2021	2021	2021	2021	2021	2021	2021	2022	2022	
Region	Germany	Sweden	Italy	United States of America	France	Italy	Germany	Finland	Germany	Japan	Italy	France	Germany	Portugal	Korea	Italy	France	Spain	North America, Europe	Poland	Slovenia	
Inclusion period	2005 to 2012	2008 to 2014	2007 to 2015	2014-15 2011-13	2010	2010 to 2012	2008 to 2016	2008 to 2017	2007 to 2012	2013-16 2003-11	2008 to 2015	2008 to 2015	2011 to 2012	2009-17 2009-16	2011 to 2019	2010 to 2018	2010 to 2019	2011 to 2020	2007 to 2019	2015 to 2019	2013 to 2019	
Numbers of centres	1	1	1		27	93	1	5		6	27	1	92	1	1	9		2		3	1	
Risk profile	High	High	High	High and intermediate	High	Low and intermediate	Low	Intermediate?	High	Intermediate	Intermediate	Intermediate	Intermediate	Low	High	Intermediate-high		Low		Low	Low	
Population size (full cohort)	52 (167)	166 (2883)	68 101	17910 22618	1334 6695	1911 5707	431 341	689 1311	419 722	338 237	486 481	104 48	4157 9066	119 544	254 66	1002 443	22380 28000	481 325	202 598	629 1765	126 175	
Propensity score matched cohort	40 40	166 125	40 40	4732 4732	799 799	650 650	109 109	308 308	216 216	153 153	291 291	48 48	1820 1820	79 79	62 62	172 172	9297 9297	171 171	"156 156	"329 593"	"53 53"	
Longest followup, years	4	11	9	1	5	5	5	4	6	2	5	2	5	9	1	7	3	3	5	6	9	
Patient's characteristics																						
Age, years	81 ± 4 80 ± 3	80 ± 9 78 ± 6	87.6 (85-92.6) 86.7 (85-91.6)	81 (77.85) 82 (77.85)	81 (76-85) 81 (77-85)	80.5 ± 6.2 80.3 ± 5.1	75.9 ± 8.4 74.4 ± 7.5	78.8 ± 6.9 79.0 ± 5.3	78.3 ± 5.2 78.2 ± 4.6	86 ± 2.8 83 ± 2.6	81 ± 6 80 ± 5	82.6 ± 5.75 79.54 ± 5.95	77.96 (6.1) 78.03 (5.09)	81 ± 8 79 ± 4	76.8 ± 6 75.5 ± 5.3	79.1 ± 7.4 80.9 ± 5.1	79.6 ± 7.3 79.4 ± 5.8	77.4 ± 8.4 78.0 ± 5.7	79.5 ± 8 79 ± 6.7		83.8 ± 2.6 83.8 ± 2.6	
Male, n (%)	29 (73) 29 (73)	84 (51.2) 79 (63.2)	18 (45) 13 (32.5)	2476 (52.3) 2454 (51.8)	427 (53.4) 434 (54.3)	267 (41.1) 263 (40.5)	45.9 (50) 45.9 (50)	148 (48.1) 143 (46.4)	100 (46.3) 111 (51.4)	47 (31) 44 (29)	121 (41.6) 121 (41.6)	24 (50) 27 (56.2)	872 (48.9) 884 (48.6)	29 (36.7) 41 (51.8)	20 (32.3) 24 (38.7)	74 (43) 67 (38.9)	0 (0) 0 (0)	62 (36.3) 64 (37.4)	90 (57.7) 85 (54.5)	124 (37.7) 261 (44)	25 (47.2) 22 (41.5)	
BMI (kg/m^2^)	27 ± 5	27 ± 4				26.5 ± 4.8 26.9 ± 4.5	27.1 ± 5.9 27.8 ± 5	28.1 ± 5.2 28.0 ± 5		22.2 ± 3.5 22.4 ± 3.7	26.2 (23-29.6) 26.6 (24-30.5)	26.06 ± 4.69 24.92 ± 3.76	28.09 (5.26) 28.14 (4.94)	27 (24-29) 27 (24-30)	24.9 ± 3.4 24.9 ± 3.3	26.7 ± 3.4 26.3 ± 2.9		29.2 ± 7.2 29.3 ± 5	27.1 ± 4.6 26.9 ± 5	28 (25-32)	29 (26-32)	
STS-PROM	11.1 ± 2.8	10.4 ± 3		5.5 (4.2-8.0)	5.8 (4.2-8.6)			3.5 ± 2.2 3.5 ± 2.8		6.2 (4.6-9.3) 4.7 (3.4-6.3)	6 (4.1-8)	6 (4.2-7.9)	4.46 (3.27)	4.58 (3.79)				2.6 (1.8-3.3) 2.8 (1.8-3.2)	5.8 ± 5.1 5.7 ± 4.3		"	2.7 ± 0.8 2.6 ± 0.8
Logistic EuroSCORE, %	23 ± 15	20 ± 14	21.6 (5-63)	18.7 (5.1-62.8)					8.7 ± 2.7	8.8 ± 2.8	13.9 (11.4- 17.4)	13.8 (11.4-16.9)										
Logistic EuroSCORE II, %	24 ± 6	19 ± 6	3.2 (1.1-14.8)	3.2(1.4-22.6)		4.9 ± 5.1 5.1 ± 6.2	2.0 ± 0.8 2.0 ± 0.8	5.0 ± 5.2 4.9 ± 5.9				5.63 ± 1.54 6.61 ± 1.82		2.43 (1.71- 3.03) 2.11 (1.49-3)	9 (14.5) 8 (12.9)	6.1 ± 1.5 5.6 ± 2.9	3.5 ± 1 3.5 ± 0.9	1.9 (1.3-2.5)	1.9 (1.3-2.5)	2.46 ± 1.5 2.02 ± 1.21	2.9 ± 1.2 2.8 ± 1.6	
Diabetes mellitus, n (%)	17 (42) 14 (35)	40 (24) 20 (16)	6 (15)	6 (15)	155 (19.4) 176 (22)	161 (24.8) 165 (25.4)	15.6 (17.0) 22 (24.0)	93 (30.2)	87 (28.2)	42 (28) 33 (22)	60 (20.6) 61 (21)	15 (31.2) 9 (18.7)	606 (33.3) 629 (34.6)	25 (32) 23 (29)	23 (37.1) 24 (38.7)	32 (18.6) 24 (13.9)	2455 (26.4) 2465 (26.5)	59 (34.5) 52 (30.4)	72 (36.5) 63 (32)	109 (33.1) 163 (27.5)	12 (22.6) 12 (22.6)	
Chronic kidney disease, n (%)	20 (50)	16 (40)	7 (17.5)	8 (20.5)	122 (15.3)	119 (14.9)			7 (3.2) 7 (3.2)	3 (2) 3 (2)	67 (23) 52 (17.9)	1 (2.08) 1 (2.08)	64 (3.5) 61 (3.4)	18 (23) 27 (34)	26 (41.9) 25 (40.3)	69 (40.1) 62 (36)	782 (8.4)	778 (8.4)		273 (82.9)	452 (76.2)	
Dialysis, n (%)	11 (6.7)	4 (3.2)		179 (3.8)	186 (3.9)	9 (1.4)	3 (0.5)				6 (2.1)	4 (1.4)	76 (4.2) 75 (4.1)	2 (2.5) 1 (1.3)	7 (11.3)	4 (6.5)				0 (0)	2 (0.3)	
COPD, n (%)	9 (23) 8 (20)	29 (18) 19 (15)	12 (30) 9 (22.5)	1948 (41.1) 1939 (40.9)	89 (11.1) 88 (11)	154 (22.3) 141 (21.7)	10.1 (11) 10.1 (11)	65 (21.1) 62 (20.1)	20 (9.3) 19 (8.8)	34 (22) 19 (12)	69 (23.7) 63 (21.6)	6 (12.5) 8 (16.6)	200 (11) 206 (11.3)	14 (18) 21 (27)	8 (12.9) 8 (12.9)	69 (40.1) 75 (43.6)	1728 (18.6) 1665 (17.9)	22 (12.9) 28 (16.4)	33 (16.8) 35 (17.8)	23 (7) 23 (3.9)	5 (9.4) 3 (5.7)	
Peripheral vascular disease, n (%)	13 (33) 11 (27)	86 (52) 49 (39)	9 (22.5) 8 (20)	1113 (23.5) 1138 (24.0)	90 (11.3) 93 (11.6)	124 (19.1)	126 (19.4)	49 (15.9) 41 (13.3)	11 (5.1) 15 (6.9)	21 (14) 24 (16)	51 (17.5)	53 (18.2)	191 (10.5)	189 (10.4)	7 (11.3) 4 (6.5)	31 (18) 22 (12.7)	2254 (24.2) 2232 (24)	12 (7) 14 (8.2)	51 (25.9) 47 (23.9)	126 (38.3) 217 (36.6)	6 (11.3) 8 (15.1)	
Prior cerebrovascular event, n (%)	9 (23) 8 (20)	13 (7.8) 10 (8)	2 (5) 0 (0)	506 (10.7) 524 (11.1)	69 (8.6) 79 (9.9)	38 (5.8) 37 (5.7)	11.9 (13) 9.2 (10)	27 (8.8) 29 (9.4)	6 (2.8) 8 (3.7)	12 (7.8) 16 (10)	19 (6.5) 17 (5.8)	4 (8.3) 2 (4.1)	196 (10.8) 182 (10)	9 (11%) 8 (10%)	14 (22.6)	15 (24.2)	280 (3) 297 (3.2)	9 (5.3)	7 (4.1)	"15 (4.6) 30 (5.1)"	"0 (0) 1 (1.9)"	
Coronary artery disease, n (%)	33 (83)	30 (75)	15 (37.5) 23 (57.5)	2406 (51)	2440 (51.6)	128 (19.7) 127 (19.5)	30.3 (33) 29.4 (32)	102 (33.1) 97 (31.5)	104 (48.1)	104 (48.1)	112 (38.5) 105 (36.1)	17 (35.4) 8 (16.6)	702 (38.6) 711 (39.1)	19 (24%) 11 (14%)	31 (50)	27 (43.6)	3990 (42.9) 3992 (42.9)	34 (19.9)	47 (27.5)	156 (47.4) 268 (45.2)	14 (26.4) 13 (24.5)	
Previous myocardial infarction, n (%)		17 (10) 16 (13)	7 (17.5) 8 (20)	1097 (23.2) 1115 (23.6)	52 (6.5) 44 (5.5)	72 (11.1) 75 (11.5)	3.7 (4) 4.6 (5)	9 (2.9) 9 (2.9)	5 (2.3) 7 (3.2)	6 (3.9) 9 (5.9)	16 (5.5)	20 (6.9)	198 (10.9)	198 (10.9)			682 (7.3) 652 (7)	14 (8.2) 13 (7.6)	68 (34.5) 72 (36.5)	152 (46.2) 255 (43)	2 (3.8) 1 (1.9)	
Previous cardiac surgery, n (%)	40 (100) 40 (100)	85 (51.2) 42 (33.6)	5 (12.5) 1 (2.5)	1406 (29.7)	1484 (31.4)	62 (9.5)	65 (10.0)	17 (5.5) 18 (5.8)	10 (4.6) 14 (6.5)	3 (2) 3 (2)	23 (7.2) 24 (8.2)	5 (10.4) 6 (12.5)	297 (16.4)	313 (17.2)	4 (6.5) 5 (8.1)	26 (15.1)	15 (8.7)	10 (5.9)	7 (4.1)			
Previous PCI, n (%)		52 (32) 24 (19)	7 (17.5) 3 (7.5)	1233 (26.1)	1278 (27.0)	94 (14.5)	85 (13.1)	47 (15.3)	40 (13)	37 (24) 11 (7.2)	51 (17.5)	44 (15.1)	328 (18)	325 (17.9)			840 (9)	863 (9.3)				
Atrial fibrillation or flutter, n (%)			19 (47.5) 17 (42.5)	1572 (33.2) 1619 (34.2)	420 (52.6)	427 (53.4)	4.6 (5) 6.4 (7)	102 (33.1)	99 (32.1)	16 (10) 23 (15)	97 (32.3) 94 (33.3)	21 (43.7) 15 (31.2)	368 (20.2) 371 (20.4)	16 (20%) 14 (18%)	5 (8.1) 8 (12.9)	37 (21.5) 28 (16.2)	4473 (48.1) 4296 (46.2)	43 (25.2) 41 (24)	54 (27.4) 54 (27.4)	56 (17) 73 (12.3)	18 (34) 19 (35.8)	
Prior pacemaker/ ICD, n (%)								20 (6.5)	19 (6.2)			8 (16.6) 4 (8.3)	174 (9.6)	167 (9.2)			922 (9.9) 903 (9.7)	13 (7.6) 11 (6.4)	13 (8.3)	9 (5.8)		
Pulmonary hypertension, n (%)	27 (16)	10 (8)	11 (27.5)	12 (30)		88 (14.6)	88 (14.6)	165 (53.6) 160 (52)	3 (1.4)	3 (1.4)	43 (14.8)	40 (13.7)	322 (17.8)	315 (17.4)						12 (3.6) 20 (3.4)	30 (56.6) 25 (47.2)	
Left ventricular ejection fraction	48 ± 14	47 ± 12	57 58.5	55.0	55.0	53.6 ± 11.4 54.2 ± 11.2			62.2 ± 11.3 62.0 ± 10.5	61 ± 12 64 ± 13	56 (49-60) 56 (50-60)	56.6 ± 13.49 58.75 ± 10.49			62.2 ± 14.6 60.8 ± 13.8	49.6 ± 9.9 48.6 ± 7.2		60.4 ± 5.6 60.0 ±12.6	52.1 ± 13.2 52.9 ± 12.9			
Aortic valve area (cm^2^)	0.63 ± 0.29	0.68 ± 0.31	0.58 ± 0.5 0.7 ± 0.2			0.7 ± 0.3 0.7 ± 0.2				0.62 ± 0.18 0.62 ± 0.17	0.65 ± 0.18 0.66 ± 0.2	0.72 ± 0.18 0.74 ± 0.2	0.72 (0.22)	0.72 (0.21)	0.72 ± 0.17 0.72 ± 0.19				"0.66 ± 0.19 0.72 ± 0.19			
Mean gradient (mmHg)	57 ± 21 51 ± 16	44 ± 12 44 ± 14	43.8 ± 16 46.4 ± 18.8	42.0 (36-52)	42.0 (35-52)	51.0 ± 14.5 51.1 ± 15.9	43.6 ± 14 42.1 ± 16.7			55 ± 18 54 ± 18	48.4 ± 15.8 48.9 ± 17.4	51.92 ± 10.89 51.04 ± 17.20	45.42 (16.69)	46.06 (16.52)	52 ± 19.4 54 ± 17.3			48.4 ± 18.6 48.4 ± 16.4				
Intervention's characteristic																						
TAVI system, (%)	Sapien (100) NA	Sapien NA	Sapien-XT NA	CoreValve (33)	NA	CoreValve (55.1) NA	Balloonexpandable (70) NA	Sapien 3 NA		Sapien XT NA	CoreValve NA		CoreValve NA	Evolut (41) NA	CoreValve (25.8%) NA	CoreValve NA		Sapien3 (57.9) NA	BEV (60.9) NA	Evolut R (56.5) NA	Sapien XT (24.5) NA	
		Sapien XT NA	Sapien-3 NA	Sapien (67) NA		Sapien XT (44.9) NA	Selfexpandable (39)	NA			Sapien XT NA		Sapien NA	Portico (8) NA	Evolut R (40.3%)	NA		Evolut PRO (19.9) NA	SEV (39.1) NA	Sapien 3 (18.8) NA	Sapien 3 (20.8) NA	
			CoreValve NA								Acurate-TA	NA	Sapien XT NA	Sapien (28) NA	Evolut Pro (4.8%)	NA		Portico (14.6) NA	Old generation (43.6) NA	Symetis (17.3) NA	Evolut R (50.9) NA	
														Lotus (2) NA	Sapien 3 (24.2%) NA			Acurate Neo (4.7)	NA	Lotus e Portico (7.4) NA	Portico (3.8) NA	
															Lotus 4.8%)	NA						
Surgical aortic valve	NA Carpentier Edwards		NA	Magna			NA Perimount	NA Perimount Magna Ease			NA Perceval S	NA Intuity	NA Perimount	NA Mitroflow	NA Perceval (96.8)	NA Perceval		NA Perceval (100)		NA Hancock II (71.5)	NA Trifecta (9.4)	
			NA	Mitroflow			NA	Hancock					NA Hancock	NA Trifecta	NA	Intuity (3.2)				NA On-X (63.4)	NA Enable (13.2)	
			NA	Hancock II			NA	Mitroflow					NA Trifecta	NA	Sorin Crown						NA Perceval (56.6)	
			NA	Solo			NA	Trifecta					NA Epic	NA Carpentier- Edwards							NA Intuity (15.1)	
														NA Perimount								
														NA	Epic							
														NA	Mosaic							
Access site, (%)	Transapical (100) NA	Transfemoral NA	Transfemoral NA	Transfemoral (76.3) NA		Transfemoral NA	Transfemoral NA	Transfemoral NA		Transfemoral (62.1) NA		Transfemoral (81.7) NA	Transfemoral NA	Transfemoral (71) NA	Transfemoral (100) NA	Transfemoral (100) NA		Transfemoral (77.2) NA	Transfemoral (100) NA	Transfemoral (100) NA	Transfemoral (100) NA	
		Transapical NA	Transapical NA										Transapical NA	Transapical (24%) NA				Transcarotid (18.1) NA				
													Transaxillary NA	Transaortic (1%) NA				Transaxillary (3.5)	NA			
			Transaortic NA										Transaortic NA	Transaxillary (4%)	NA			Transaortic (1.2) NA				
MIC-SAVR, n (%)							NA	46.8 (51)			Mini sternotomy e mini thoracotomy							NA	75 (43.9)	NA MIC (3)	NA Mini J sternotomy (49.1)	
																					NA Mini thoracotomy (50.9)	
Concomitant CABG, n (%)				NA	1565 (33.1)			14 (4.5)	84 (27.3)													

BEV=balloon-expandable valve; BMI=body mass index; CABG=coronary artery bypass grafting; COPD=chronic obstructive pulmonary disease; EuroSCORE=European System for Cardiac Operative Risk Evaluation; ICD=implantable cardioverter defibrillator; MIC=minimally invasive cardiac surgery; NA=not applicable; PCI=percutaneous coronary intervention; SAVR=surgical aortic valve replacement; SEV=self-expanding valve; STS-PROM=Society of Thoracic Surgeons Predicted Risk of Mortality; TAVI=transcatheter aortic valve implantation

Six studies were at critical risk of bias, 15 were at moderate risk. Assessment of Domain 5 was not possible in most of the selected studies, as there is no information on missing and how missing were handled.

### Quality Assessment of Estimated Reconstructed Time-To-Event Data

No major graphical differences were shown at visual comparison between original reported KM curves and estimated KM curves. HRs estimated from RTE data were compared to HRs in the paper, when available. HRs estimated from RTE data were not different to those reported in the trials, confirming a high accuracy of the reconstructing time-to-event data method.

### Analysis of Death from Any Cause Up to Five Years

**Fig. 1:**
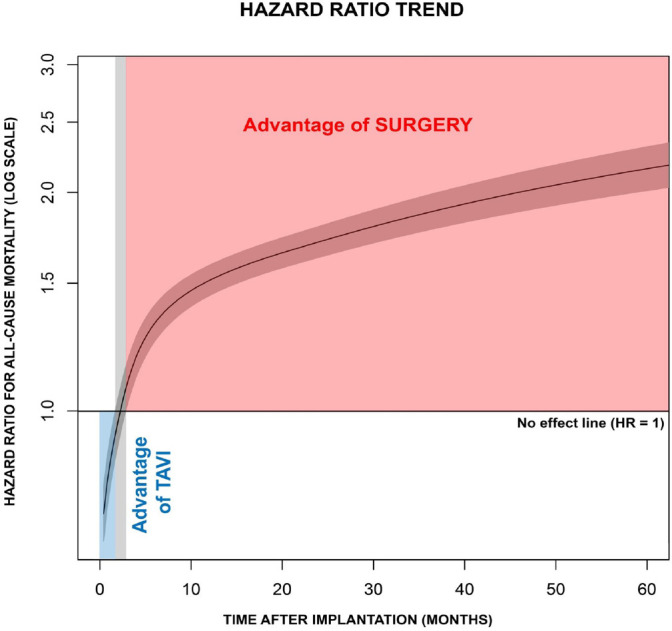
Kaplan-Meier incidence function of all-cause mortality in transcatheter aortic valve implantation (TAVI) and surgical aortic valve replacement groups. There is a strong departure from the constancy of the hazard ratio (HR). Hence, the Cox-derived HR should be interpreted with caution.

Figure 1 shows the KM estimates for all-cause mortality, based on estimated 871361.3 patient-months follow-up. The difference between TAVI and SAVR curves was significant (Log-rank *P*-value < 0.001). Surgery was associated with a survival advantage over TAVI, as demonstrated by the grouped frailty semi-parametric modeling (HR 1.41; 95% confidence interval [CI] 1.34 - 1.47, *P*-value < 0.001), with a significant heterogeneity (random parameter θ = 0.1, *P*-value < 0.001). However, the Cox model was invalidated by the strong departure from constancy of the HR, underscored by the Shoenfeld residuals and the Grambsch-Therneau test for time-invariant effect (*P*-value < 0.001), that leads to misleading effect estimation. Therefore, we proceeded with the analysis of HR trend over time.

**Fig. 2:**
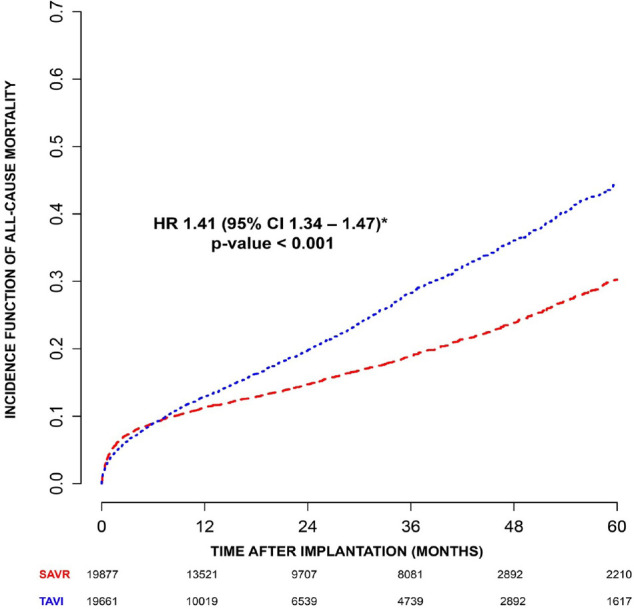
Hazard ratio (HR) trend over time for all-cause mortality of transcatheter aortic valve implantation (TAVI) vs. surgical aortic valve replacement (SAVR) estimated by fully parametric generalized survival model. CI=confidence interval.

The analysis of HR trend over time of TAVI *vs.* SAVR estimated by fully parametric generalized survival models showed that TAVI is superior to surgery limited to the first months with a steep reversal afterwards, when SAVR became clearly superior (Figure 2). After one month, the advantage of surgery increased progressively, with a two-fold hazard of death for TAVI after 40 months.

**Fig. 3:**
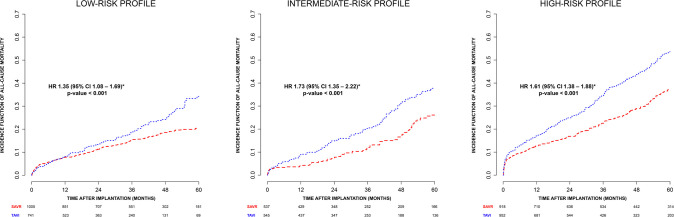
Kaplan-Meier incidence function of all-cause mortality in transcatheter aortic valve implantation (TAVI) and surgical aortic valve replacement (SAVR) groups in low-, intermediate-, and high-risk subgroups. There is a strong departure from the constancy of the hazard ratio (HR); hence, the Cox-derived HR should be interpreted with caution. CI=confidence interval.

**Fig. 4:**
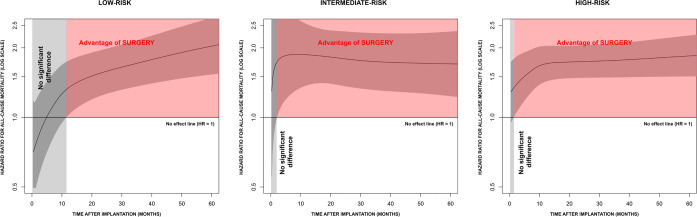
Hazard ratio (HR) trend over time for all-cause mortality after transcatheter aortic valve implantation vs. surgical aortic valve replacement estimated by fully parametric generalized survival model in subgroups of patients at low, intermediate, and high risk.

The analysis performed in the subgroups of risk confirmed data on the whole sample, although they represent only a small sample of the entire cohort, being most of the studies performed in mixed risk population. KM estimates for all-cause mortality in low-risk profiles showed a significant difference favoring surgery (HR 1.35; 95% CI 1.08 - 1.69, *P*-value < 0.001) (Figure 3), with a significant heterogeneity (random parameter θ = 0.1, *P*-value < 0.001). The Shoenfeld residuals and the Grambsch-Therneau test for time-invariant effect *P*-value was 0.02, representing a significant departure from constancy of the HR. Surgery has a five-year benefit also in intermediate risk (HR 1.73; 95% CI 1.35 - 2.22, *P*-value < 0.001; random parameter θ = 0.11, *P*-value < 0.001) (Figure 3) and high risk (HR 1.61; 95% CI 1.38 - 1.88, *P*-value < 0.001; random parameter θ = 0.001, *P*-value 0.5) (Figure 3). The HR trends in the subgroups of risk are influenced by the lower sample size. However, they corroborate the hypothesis of the benefit of surgery at five years (Figure 4).

## DISCUSSION

The current meta-analysis of RTE data from propensity score matching studies compares the effectiveness of TAVI *vs.* SAVR on mid-term all-cause mortality, aiming to evaluate the generalizability of TAVI indications. The main findings of this study are:

1. There is a significant difference in all-cause mortality at five years between TAVI and SAVR, favoring surgery.
2. TAVI maintains a survival advantage only in the perioperative period.
3. In the low-risk subgroup, TAVI is not superior to surgery, even in the first month after the procedure.

Our results on pooled RWD produced evidence of effectiveness at follow-up of TAVI and SAVR in real-world practice settings that differ from the efficacy that emerged by RCTs, raising concern about their external validity and the risk related to the indication creep^[13]^. All RCTs have demonstrated superiority or non-inferiority of TAVI compared to surgery, at least at one or two years, but on top of concerns related to the high risk of bias that can affect internal validity^[10]^, they are designed on very selected subgroups of the population, as can be concluded by the several inclusion/exclusion criteria. The study selection is increasingly narrowed with the decrease of the risk profile of the trials’ cohort. A low-risk profile merges very different patients with diverse life expectancies and/or ages. Nonetheless, considering that aging is accompanied by an increase in chronic comorbidities, the elderly are likely to have an intermediate or high-risk profile, and the quote of elderly patients with no comorbidities should be limited and scarcely represented in the low-risk cohorts. Instead, only 8% of the patients were younger than 65 years in the PARTNER 3 and EVOLUT LR trials, and the mean age of all low-risk trials is higher than 73 years (79 years in the NOTION trial)^[5-7,13]^, corroborating the hypothesis of high selection bias of the study groups.

The generalizability of the findings from the highly selective RCTs to broader populations (indication creep) without supporting data may lead to unexpected outcomes^[44]^. The results of the pooled RWD presented in this meta-analysis do not support the non-inferiority of TAVI shown by all RCTs and are also not concordant with meta-analyses on RCTs^[1-8,45-48]^. The survival advantage of TAVI in the first 24 months after implantation in RCTs in a methodologically similar meta-analysis is not corroborated by pooled RWD, as it runs out in the first month and reverses with a growing difference in mortality favoring surgery^[45,46]^. Other marked discrepancies are highlighted in the low-risk group, which is most likely to suffer the consequences of unsupported indication creep. A recent meta-analysis found no differences after one year in main outcomes between TAVI and SAVR in low-risk and high-risk patients, while the sub-analysis in the low-risk group of the present meta-analysis confirms that TAVI is associated with an increasingly worse all-cause mortality at five years and shows that there is also no advantage in the first months after the procedure^[48]^. The absence of a TAVI advantage in the perioperative period may be simply related to the lower sample size, although it might also be justified by a less pronounced effect of surgical invasiveness in low-risk patients leading to smaller perioperative differences between treatments. The outcomes of this meta-analysis on RWD pose a serious threat on the external validity of the existing first-line evidence that demonstrated the efficacy of TAVI especially in low-risk patients and drove the guidelines towards expanding the indication for TAVI to all categories of risk.

RWD has been reevaluated as a credible source of information, as it can provide evidence that informs patients, physicians, and regulators on the effects of an intervention outside the narrow confines of the research setting^[11,12,15]^. RCTs may exclude most patients seen in routine care; multimorbidity has a median exclusion proportion over 90% in RCTs, and large evidence gap has been noted for cardiovascular disease and psychiatric conditions, with an undisputed threat for their generalizability^[49]^. RWE may help regulators provide reliable information on treatment’s benefits and risks in heterogeneous clinical settings, such as vulnerable patients^[12]^. RWD is useful to improve the management of rare conditions, guarantee lower costs, and may also allow for longer follow-up, optimizing the detection of adverse events^[11,12,15]^. Companies and payers use systematic collection of RWD to monitor the effectiveness of new products and to bolster formulary positioning. Advances in methodologies applied to RWD as well as the availability of higher-quality larger datasets have bolstered their routine employment. Regulatory bodies, such as the FDA and the European Medicines Agency (or EMA), recognized the role of RWE in supporting their assessments and decision-making. RWD holds an intrinsic high risk of bias that can be reduced but not nullified and cannot substitute for the RCT design, which remains the gold standard for assessing the efficacy of a treatment. Nonetheless, observational studies can provide complementary information on treatment’s effectiveness and should be employed to supplement first-level evidence in health care decision-making^[15]^.

The detailed explanation of the different TAVI/SAVR effectiveness at five years is far beyond the aims of the present study. Durability of the prosthesis, paravalvular leaks, and a higher incidence of pacemaker implantation have been considered potential factors affecting mid-term outcomes^[39]^. Newer prostheses are claimed to have better performance and a lower incidence of structural and non-structural valve deterioration, although there is limited evidence to support these arguments.

### Limitations

Our pooled meta-analysis of RTE data holds intrinsic limitations. The duration of follow-up has been limited to five years as only a few patients had a longer follow-up. Most included studies have been performed in a mixed population (88%) of risk and treatments. Hence, evaluation on different devices is not feasible. A subgroup analysis should be taken with caution as the sample size is small. Moreover, the potential impact of comorbidities on both heterogeneity and outcomes in individual patients cannot be extrapolated.

## CONCLUSION

In the real-word setting, TAVI is associated with a significant progressively worse incidence of all-cause of death compared to surgery and maintains a survival benefit only in the first month after implantation. However, in the subgroup of low-risk patients, the initial advantage is not evident. The results of this meta-analysis of propensity score matching studies comparing TAVI and SAVR show that TAVI effectiveness may not reflect the efficacy demonstrated by RCTs and pose a serious threat to their external validity.

## Data Availability

Data underlying the meta-analysis are retrieved from published RCTs and hence are already available in literature; no unpublished data were employed. However, the collected data underlying this article will be shared on reasonable request to the corresponding author.
